# The Genome of *Apera spica-venti*: A Major Grass Weed

**DOI:** 10.1093/gbe/evaf096

**Published:** 2025-05-22

**Authors:** John Lemas, Jevgenija Ņečajeva, Jacob Montgomery, Sofia Marques-Hill, Victor Llaca, Kevin Fengler, Lena Ulber, Dagmar Rissel, Josef Soukup, Kateřina Hamouzová, Fatemeh Abdollahi, David Nelson, Todd A Gaines, Eric Patterson

**Affiliations:** Department of Plant Soil and Microbial Sciences, Michigan State University, East Lansing, MI, USA; Faculty of Medicine and Life Sciences, University of Latvia, Riga, Latvia; Department of Plant Soil and Microbial Sciences, Michigan State University, East Lansing, MI, USA; Department of Agricultural Biology, Colorado State University, Fort Collins, CO, USA; Department of Agricultural Biology, Colorado State University, Fort Collins, CO, USA; Genomics Lab, Corteva Agriscience, Johnston, IA, USA; Genomics Lab, Corteva Agriscience, Johnston, IA, USA; Institute for Plant Protection in Field Crops and Grassland, Julius Kühn Institute (JKI), Braunschweig, Germany; Institute for Plant Protection in Field Crops and Grassland, Julius Kühn Institute (JKI), Braunschweig, Germany; Department of Agroecology and Crop Production, Faculty of Agrobiology, Food and Natural Resources, Czech University of Life Sciences, Prague, Czech Republic; Department of Agroecology and Crop Production, Faculty of Agrobiology, Food and Natural Resources, Czech University of Life Sciences, Prague, Czech Republic; Department of Agricultural Biology, Colorado State University, Fort Collins, CO, USA; Department of Microbiology, Immunology, and Biochemistry, University of Tennessee Health Science Center, Memphis, TN, USA; Department of Agricultural Biology, Colorado State University, Fort Collins, CO, USA; Department of Plant Soil and Microbial Sciences, Michigan State University, East Lansing, MI, USA

**Keywords:** genomes, *Apera spica-venti*, grass, weeds, herbicide

## Abstract

*Apera spica-venti* (loose silky bent, or common windgrass) is a diploid grass-weed endemic to Europe and north Asia that has spread to the United States and Canada. This species has become a major grass weed in winter cereals, especially in eastern Europe mainly through the evolution of target site and nontarget site resistance mechanisms. The scientific community currently lacks genomic resources to understand herbicide resistance evolution in this plant and therefore resistance is hard to diagnose and treat. To remedy this, we generated two reference haplome assemblies through phased genome assembly. Haplome 1 consists of 37 scaffolds with a total length of 4.06 Gbp and an N50 of 206.5 Mbp, while haplome 2 resulted in 34 scaffolds with a total length of 3.99 Gbp and an N50 of 270.1 Mbp. Both haplomes represent over 87% of the flow cytometry estimated genome size of 4.622 Gbp per 1C. Gene annotation was performed via a modified Maker pipeline resulting in 44,208 and 43,844 genes for haplomes 1 and 2, respectively, and capturing 90% of BUSCO annotated transcripts. Repeat analysis identified greater than 800,000 transposon elements spanning 2.3 Gbp of the genome and an average distance between genes of over 90 kbp. This reference genome addresses the lack of genomic resources and aims to better understand basic weed biology, ecology, and herbicide resistance evolution.

**Significance**

This genome provides the scientific community with a genomic resource for this economically important grass weed and serves as the first genomic resource for the *Apera* genus. Analyses presented in this report, including the identification of important genes in the detoxification of herbicides, will serve as a steppingstone for future omics analysis of weed biology, ecology, and evolution.

## Introduction

Loose silky bent or common windgrass (*Apera spica-venti* (L.) P. Beauv, syn. *Agrostis spica-venti* L., *Anemagrostis spica-venti* Trin., *Apera maritima* Klokov) belongs to Pooideae, the largest subfamily of the grass family (Poaceae). Pooideae includes major cereal crops (wheat, barley, rye, oat) as well as many forage grasses and grass weed species; however, the genus *Apera* is rather small with only about five species currently recognized (Watson and Dallwitz 1992)*. Apera spica-venti* is native to Europe and North Asia (Soukup et al. 2006) but through a series of invasion events, it has spread to the northeastern United States and Canada (Warwick et al. 1987). In Europe, this weed species is a major problem in several central and eastern European and Scandinavian countries (Soukup et al. 2006; Melander et al. 2008). A closely related species *Apera interrupta* (*L*.) Beauv. (interrupted windgrass), originating from southwest Asia, is also a problematic invasive weed in the northwestern United States but found in many other areas including much of Canada (Northam and Callihan 1992).

*Apera spica-venti* is diploid (2*n* = 14), outcrossing species, predominantly self-incompatible, with high levels of genetic variability within populations (Warwick et al. 1987). It primarily exhibits a winter annual growth habit and is one of major grass weeds in winter cereals. A single plant of *A. spica-venti* can produce up to 5,600 seeds (Bitarafan and Andreasen 2020). Seeds exhibit low dormancy and mostly germinate in autumn soon after they are shed (Cici and Van Acker 2009). Some seedlings, however, can emerge in early spring and infest spring crops as vernalization is not required for floral induction in this species (Warwick et al. 1987). Crop rotations with a high proportion of winter crops and non-inversion tillage methods that incorporate seeds in the upper soil layers favor infestation with *A. spica-venti* and up to 30% cereal yield loss can be caused at weed density of 200 plants m^−2^ (Melander et al. 2008). The economic risks posed by *A. spica-venti* are exacerbated by its evolved resistance to several herbicidal sites of action, which complicate chemical control in this species.

Outcrossing and high genetic variability increases the risk of evolution and spread of herbicide resistance in *A. spica-venti*. The International Herbicide-Resistant Weed database lists 18 unique cases of herbicide resistance in *A. spica-venti* in 11 different European countries, including five unique cases of multiple resistance with two to three modes of action (Košnarová et al. 2021; Heap 2024). Four target site resistance (TSR) mutations associated with resistance to acetolactate synthase (ALS) inhibitors were identified in *A. spica-venti* resistant to sulfonylureas in Czech Republic (Hamouzová et al. 2014). Research in Denmark revealed that resistance to ALS inhibitors was caused by nontarget site resistance (NTSR), such as mechanisms of metabolic detoxification involving Cytochrome P450 enzymes (Cyp450), while both target and nontarget site resistance mechanisms conferred resistance to acetyl-CoA carboxylase (ACC) inhibitor (Babineau et al. 2017b). Resistance to ALS inhibitors can evolve simultaneously as target site mutations and NTSR (Rissel and Ulber 2018). Another study from the Czech Republic has shown that multiple resistance to three modes of action can result from the combination of TSR and NTSR (Košnarová et al. 2021). Most recent studies involving transcriptome analysis revealed more mechanisms that potentially confer NTSR to *A. spica-venti* plants. A published reference transcriptome of *A. spica-venti* contains 74,724 transcripts consisting of total 54,846,111 bp (Babineau et al. 2017a). Analysis of de novo transcriptomes of sensitive and resistant plants after treatment with ACC inhibitor showed that genes from several pathways associated with NTSR, such as ABC transporters, Cyp450, as well as 3-ketoacyl-CoA synthase 12-like, UDP-glycosyltransferases and glutathione S-transferases were differentially expressed in resistant and sensitive plants (Wrzesińska-Krupa et al. 2023). Further research of genetic regulation of NTSR is required to develop new screening methods to allow early detection of herbicide resistance risks and to better understand underlying mechanisms of resistance evolution. The completion of the *A. spica-venti* genome as part of the International Weed Genomics Consortium will be instrumental in fully understanding genes involved in NTSR as well as other complex weedy traits as researchers continue to find new ways to control and mitigate the damage caused by this critical weed species. (Montgomery et al. 2024).

## Results and Discussion

### Telomere-to-Telomere Genome Assembly

**Fig. 1.**
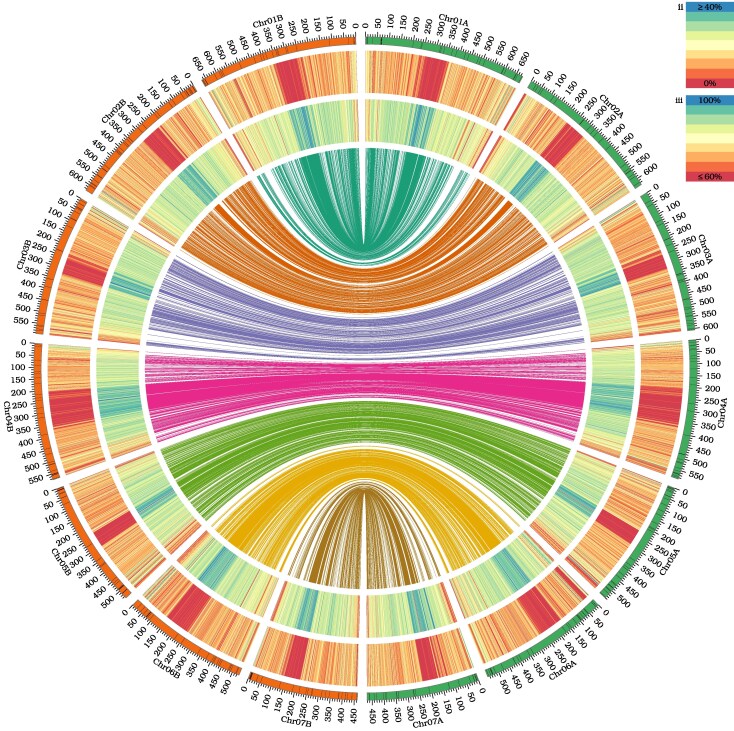
Genomic features of *Apera spica-venti* haplome 1 (right, chromosomes 1 to 7 labeled with A) and haplome 2 (left, chromosomes 1 to 7 labeled with B) assemblies. Circos plot depicts: from outer track to inner track: (i) number and length (Mbp) of chromosomes with black representing gaps; (ii) gene density across the chromosomes, with blue representing gene-rich regions, yellow representing intermediate regions, and red representing gene-poor regions; (iii) repetitive element density along chromosomes, with blue representing repeat-rich regions, yellow representing intermediate regions, and red representing repeat-poor regions; and (iv) inner ribbons represent regions of similarity (mapping quality = 60 and alignment length > 100 kb) between haplomes. Ribbons colored by which chromosome in the reference haplome each alignment lands on. Window size of 1 Mbp for ii and iii.

The average genome size for four biological replicates was 4.622 Gbp per 1C (±48 Mbp), as determined by flow cytometry. The final assembled genome size of the two haplomes of *A. spica-venti* was 4.06 Gbp and 3.99 Gbp for the reference and alternative, respectively (Table 1). The final assembly of both haplomes was scaffolded into seven chromosome-sized pseudomolecules, representing the predicted seven chromosomes of this species (Montgomery et al. 2024). Assembly methods reduced the need for manual curation and gap filling. Visualization of the haplome assemblies shows pockets of concentrated repetitive sequence near the middle of each chromosome that correlate with reduced gene densities (Fig. 1). The pseudomolecules had 276 and 298 gaps in the reference and alternative assemblies, respectively (Table 1). These gaps seem to be primarily comprised of hard to assemble, AT rich repeat sequence, which are generally gene-poor regions, and are also co-clustered with the highly repetitive centromere locations (Fig. 1) (Talbert and Henikoff 2020). The discrepancies between predicted genome size and assembled genome size seem to be extremely long regions of AT rich content that are unable to be resolved and reside in the gaps in the assembly. The two haplomes have retained a high degree of synteny along their entire lengths (Fig. 1). Chromosomes 2 and 5 did not display obvious structural differences; however, inversions were detected in chromosomes 1, 3, 4, 6, and 7 (supplementary fig. S2, Supplementary Material online). In the reference assembly, chromosomes 1, 2, 3, and 6 contain telomere sequence on both sides, while chromosomes 4, 5, and 7 have telomeric sequence on either the left or right side (supplementary table S1, Supplementary Material online).

**Table 1:** Assembly and annotation statistics for *A. spica-venti* reference and alternative assemblies

Assembly		Reference		Alternative
Total length of assembly		4,060 Mbp		3,990 Mbp
Chromosome 1		668.7 kbp		678.0 kbp
Chromosome 2		626.9 kbp		629.8 kbp
Chromosome 3		606.6 kbp		593.7 kbp
Chromosome 4		586.3 kbp		574.2 kbp
Chromosome 5		538.6 kbp		540.9 kbp
Chromosome 6		529.9 kbp		549.3 kbp
Chromosome 7		462.7 kbp		471.4 kbp
HiC phased contigs		245		239
Phased contig N50		44.3 Mbp		42.8 Mbp
Bionano maps		61		58
Map N50		178.3 Mbp		135.8 Mbp
Hybrid scaffolds		37		34
Hybrid scaffold N50		206.5 Mbp		270.1 Mbp
Number of gaps		276		298
Average gap length		8.3 kbp		14.3 kbp
Gap N50		88.4 kbp		112.1 kbp

BUSCOs	Gen.	Trans.	Gen.	Trans.
Complete (#)	1,599	1,452	1,593	1,462
Complete (%)	99.1	89.9	98.7	90.6

Additional BUSCO scores are reported in supplementary table S2, Supplementary Material online.

### Annotation and Repeat Analysis

Genome annotation was performed using IsoSeq reads and a modified Maker pipeline that included identifying and masking repeats using RepeatModeler and Repeat Masker (Cantarel et al. 2008; Flynn et al. 2020). Predicted IsoSeq transcripts mapped at a rate of 98% for both haploid assemblies. After Maker, this pipeline predicted 44,208 and 43,884 genes in the reference and alternative genomes, respectively. BUSCO scores on the genome and predicted transcripts are 99.1% and 89.9%, respectively, indicating that the genome captures almost the entire gene space (Manni et al. 2021); however, ∼10% of the predicted genes are absent from the transcriptome (supplementary table S2, Supplementary Material online). Surprisingly, there are relatively few duplicated genes (∼4.5%) indicating that a large-scale genome duplication event has not occurred in this lineage recently, as we would expect a higher rate of gene duplication. Given the ∼4 Gbp length of the *A. spica-venti* genome, we were curious what is driving the large genome size. The average distance between genes in the genome can be calculated at >90 kbp, which means that a vast majority of the genome is noncoding or repetitive DNA. There are only a few, relatively small, regions of the genome where gene density is high, at the ends of some chromosome arms, otherwise it is sparse (Fig. 1). When we look at the predicted output from RepeatModeler, it characterizes >800,000 LTR transposon elements, spanning over 2.3 Gbp of the genome; 163,576 of these elements are classified as Ty1/Copia elements with 624,817 being classified as Gypsy/DIRs. Unclassified and interspersed repeats also make up a large portion of the genome at 919 Mbp and 3.46 Gbp, respectively (supplementary table S3, Supplementary Material online). It seems that the relatively large genome size of *A. spica-venti* is due to the proliferation of repetitive DNA elements.

### Cytochrome P450 Identification Results

In *A. spica-venti*, a total of 513 P450 genes were identified and named according to the Standardized Cytochrome Nomenclature Committee. These genes were grouped into 42 families and 102 subfamilies (see supplementary table S4, Supplementary Material online). Of the identified genes, 421 were found to be full-length, with lengths ranging from 350 to 558 amino acids, whereas 92 were classified as fragments, containing fewer than 350 amino acids (supplementary table S4, Supplementary Material online). For the P450 clan classification, the 421 full-length genes were used to generate a neighbor-joining (NJ) tree. The genes were categorized into two main groups: A-type, which includes only clan 71, and non-A-type, which encompasses multiple clans including 710, 85, 711, 86, 97, 72, 51, 727, and 74. Some clans, such as 51, 74, 727, 97, 710, and 711, each comprised only one gene family, while other clans like 71, 72, 85, and 86 represented multiple gene families (supplementary fig. S1, Supplementary Material online).

### Conclusion

The genome of *A. spica-venti* will be a valuable resource for weed scientists who are trying to control its spread and better understand its biology. With this resource, they will be able to design molecular assays, identify genes of interest, and ask more detailed questions concerning its genetics and molecular biology. This genome also represents the first genome assembly in the small grass genus *Apera*, making it an asset for grass geneticists who wish to better understand the evolution and relatedness of grasses, especially those within the bamboos, rice, and Pooideae (BOP) clade. Furthermore, this genome forwards the goal of the International Weeds Genomics Consortium to equip the weed science community with high quality reference genomes in their global effort to expand molecular, genomic, and basic biological knowledge of important agronomic pests (Montgomery et al. 2024).

## Materials and Methods

### Sample Preparation, DNA Extraction, and Sequencing

All materials for sequencing were gathered from one individual obtained from an herbicide-susceptible *A. spica-venti* population collected from an organically farmed field near Braunschweig (Germany) in 2012. There is no specific legislation on access to genetic resources as well as benefit-sharing in Germany. Accordingly, regulations according to the Convention on Biological Diversity and the Nagoya Protocol are not applicable here.

The estimated genome size was evaluated at the Flow Cytometry Facility of the Iowa State University Office of Biotechnology using *Zea mays* as an internal standard and fresh young leaves from *A. spica-venti* individuals. Fresh tissue was used for Bionano optical mapping, and flash-frozen young tissue was used for PacBio HiFi and HiC chromatin conformation. Flash-frozen tissue of roots, stems, young leaves, and flowers was used for RNA extraction and PacBio IsoSeq. Flash-frozen tissue and RNA samples were shipped on dry ice and Bionano tissue samples were transported at 4 °C to the Genome Center of Excellence at Corteva Agriscience for DNA extraction, library preparation, and sequencing as described by Lemas et al. (2025) resulting in this assembly.

### Optical Genome Mapping Assembly

Ultra-high molecular weight (uHMW) DNA was isolated using a modified version of the Bionano Genomics Plant Tissue DNA Isolation Base protocol Bionano (PN:80003; Bionano Genomics). Young leaf tissue was collected from a single individual and shipped in moist germination paper. Approximately 500 mg leaf tissue was immersed in a 2% formaldehyde Bionano fixing solution for 20 min, washed, chopped, and homogenized using a Qiagen TissueRuptor probe in homogenization buffer. The homogenate was passed through 100- and 40-µm cell strainers to remove cell debris, and the pass-through sample was centrifuged at 2,000 × *g*. The pelleted nuclei were resuspended in homogenization buffer and subject to two cycles of low-speed centrifugation (100×) to remove additional debris and other solids. Supernatant containing the nuclei was recovered every time, and then centrifugated at 2,000 × *g*. The pelleted nuclei were resuspended in Bionano Washing solution, centrifugated at 2,000 × *g*, and resuspended before being embedded in low melting point agarose. The nuclei were lysed by treating the resulting agarose plug with proteinase K and RNase A as described previously (Hufford et al. 2021), washed in Wash Buffer and TE buffer. uHMW DNA was eluted from agarose by melting the plug at 65 °C, incubating in the presence of agarose at 43 °C for 45 min, and finally performing drop dialysis against TE.

Approximately 1 µg purified, highly viscous DNA sample was processed according to the Bionano Direct Label and Stain (DLS) protocol and loaded into a Bionano chip, as per manufacturer's protocol (PN 80005). Resolved molecules were imaged and digitized using a Saphyr ICS. Data visualization, map assembly, and hybrid scaffold construction were performed using Compute Servers using Bionano Access (v1.7) and Bionano Solve (v3.7_10192021_74_1). In total, 2,820.34 Gbp in molecules with a minimum of 150 kbp and N50 length of 304.13 kbp was sized selected to generate a 1,156.62 Gbp in molecules with a minimum of 350 kbp and N50 length of 518.25 kbp. This filtered set included 2,154,537 molecules, which were assembled initially without phasing, using standard non-haplotype, no-CMPR-cut parameters without extend-split parameters, leading to a 7,977.96 Mbp assembly consisting of 549 maps with map N50 of 32.7 Mbp.

Haplotype-specific molecule baiting yielded two subsets of molecules: A (1,029,460) and B (1,022,473), with N50 lengths of 524 and 516 kbp, respectively. The phased (haplotype aware) assemblies had an increased contiguity and reduced total length. Haplotype A Bionano assembly consisted of 90 maps with an N50 map length of 178.38 Mbp and total length of 4.04 Gbp, while the Haplotype B Bionano assembly had 89 maps, N50 map length of 135.83 Mbp and total length of 4.01 Gbp.

### Hi-C Seq

Approximately 500 mg frozen young leaf tissue was ground in a mortar with liquid nitrogen to a fine powder, crosslinked with formaldehyde and used to prepare a Hi-C Seq library using the Proximo Plant system (Cat. KT3040; Phase Genomics, Seattle, WA), as per manufacturer's protocol. Library was cleaned, size-selected, and quantified/qualified using Qubit 3.0 and Agilent Tapestation. The library was sequenced in an Illumina NovaSeq 6000 system collecting 150 bp. A total of 1,091,147,576 paired end clusters were collected for a total of 327,344,272,800 bp.

### HiFi Sequencing

DNA was isolated from approximately 1 g of frozen young leave tissue using the Nucleobond HMW DNA kit as per manufacturer's protocol (part 740160; Macherey-Nagel) with a few modifications, quantified using Qubit 3.0 and assessed by DNA pulsed-field electrophoresis in an Agilent Femto system. The DNA with a center of mass of above 100 kbp was sheared using a Megaruptor 3 (Diagenode) set at speed 34, resulting in a center of mass of approximately 15 kbp as determined by Femto. The sheared DNA was used to prepare a SMRTBell HiFi Sequencing library according to the standard PacBio Procedure & Checklist using the Express Template Prep Kit 2.0 (PN: 101-853-100). Library was size-selected using the 15 to 20 kbp High Pass 75E protocol in a PippinHT System (Sage Science; PN: HPE-7510). The sized SMRTbell library was bound to the sequencing polymerase enzyme using the procedure generated by the SMRT Link Sample Setup. Six SMRTCells were prepared and a total combined of 586.8 Tbp total read length, ultimately processed into a total of 226.350 Gbp HiFi CCS total length. The 107.9 million HiFi reads, generated using the CCS algorithm with an average of ten passes, had an average read length of 17.897 kbp and median Q32 read quality.

### Assembly Pipeline

PacBio HiFi and Hi-C sequencing data were used as inputs to generate a primary phased assembly with Hifiasm (v0.24) (Cheng et al. 2021). The resulting assemblies were aligned to Bionano optical maps using Bionano Solve (v3.7) to assign molecules to each haplotype. Hybrid scaffolds were constructed by aligning the phased assemblies with the optical maps using Bionano solve (v3.7). The Bionano hybrid scaffolds served as a reference for Hi-C reads alignment done by Bwa mem (v 0.7.17) (Li 2013) called by Juicertools (v1.19.2) (Durand et al. 2016). The resulting Hi-C contact maps were then visualized with Juicebox (v1.11.08; Broad Institute and Aiden Lab), which enabled manual curation and scaffolding of hybrid scaffolds into chromosome-scale assemblies (super-scaffolds or pseudomolecules). During this process, scaffolds were joined by inserting 100 Ns at each junction. Finally, pseudomolecules were ordered by size and sequentially named from largest to smallest.

### Haplotype-Resolved Assembly and Scaffolding

The phased map sets produced two separate hybrid scaffold sets for haplomes 1 and 2. Haplome 1 was captured in 37 scaffolds with total length of 4.06 Gbp and N50 length of 206.5 Mbp. Haplome 2 consisted of 34 scaffolds with total length of 3.99 Gbp and N50 length of 270.1 Mbp.

### Genome Annotation

Gene annotation utilized public databases along with gathered PacBio IsoSeq transcriptomic data as described by Lemas et al. (2025). To summarize, IsoSeq reads were generated from leaf, stem, root, and floral tissues. Assemblies were softmasked using RepeatModeler (v.2.0.2) (Flynn et al. 2020) and RepeatMasker before IsoSeq read alignment with Minimap2 (Li 2018). Protein sequences were obtained from the *Lolium perenne* (Nagy et al. 2022) genome for use as a control in Maker (Cantarel et al. 2008) resulting in final annotated features. Public databases including InterPro, UniRef, and NCBI were used to assign functional annotation information. Annotated features were used to generate *A. spica-venti* protein sequences for BUSCO (v4.0.2) (Manni et al. 2021) analysis using GffRead (v.0.12.7) (Pertea and Pertea 2020) The embryophyta_obd10 BUSCO database was used for both predicted (genomic) and annotated (proteomic) analyses as reported in supplementary table S2, Supplementary Material online.

### Genome Analysis

RepeatModeler (v.2.0.2) (Flynn et al. 2020) was used to identify repetitive elements across the genome, and assembly statistics for the seven assembled chromosomes were generated through Assemblathon (Pearl v5.16.3) (Earl et al. 2011) and are displayed in Table 1. Gene density was calculated by counting the number of genes in 1 Mbp windows across each haplome. RepeatModeler2 (v2.0.3) was used to detect repetitive elements across each haplome, and the resulting gff file was used to determine the percentage of bases within 1 Mbp windows that were contained within repetitive elements (Flynn et al. 2020). Minimap2 (v2.17; -x asm5) was used to align the chromosomes of the alternate haplome to the chromosomes of the reference haplome (Li 2018). The resulting alignments were filtered to only retain alignments with mapping quality of 60 and alignment length of at least 100 kbp. Circos (v0.69.9) was used to plot gene and repeat density along with regions of synteny (Krzywinski et al. 2009).

### Identification, Naming, and Classification of Cytochrome P450 Genes

The identification of P450 genes was carried out following the method described by Lemas et al. (2025). Briefly, the annotated protein sequences of *A. spica-venti* were searched using the InterPro codes “IPR001128” and “IPR036396.” The resulting CYP450 candidates were named in accordance with the guidelines established by the Standardized Cytochrome Nomenclature Committee (http://drnelson.uthsc.edu/CytochromeP450.html). To perform phylogenetic tree analysis and clan classification, we aligned 421 full-length CYP450 protein sequences from *A. spica-venti* using Clustal W. The NJ phylogenetic tree was generated with 1,000 bootstrap replicates using MEGAX software. The final tree was visualized through the ITOL9 web server (https://itol.embl.de/).

## Supplementary Material

evaf096_Supplementary_Data

## Data Availability

Assemblies for both the reference (haplome 1) and alternative (haplome 2) haplomes are available on Weedpedia (https://weedpedia.weedgenomics.org/) and at NCBI under BioProjects PRJNA1171856 and PRJNA1171857; genome accessions: JBMEVH000000000 and JBMEVI000000000. Sequencing reads and other data used in the assembly and annotation are available at NCBI under BioProject PRJNA1171852; BioSample SAMN44257079; SRA accessions SRR30961305 (Hi-C data), SRR30961306 (IsoSeq data), and –SRR30961307 (Genomic HiFi data); and BioNano.cmap Supplementary File SUPPF_0000005647. Reviewers can access the genome assemblies and annotations at Weedpedia using the following login information: website: https://weedpedia.weedgenomics.org/; user name: reviewer; password: HvttnT8ptN8M34Uy!.
