# Supplementary material for: The Genome of Apera spica-venti: A Major Grass Weed
Source: Genome Biol Evol. 2025 May 22;17(6):evaf096. doi: 10.1093/gbe/evaf096 (PMC12127512; doi:10.1093/gbe/evaf096)
Supplement: evaf096_Supplementary_Data [file evaf096_supplementary_data.docx]

**Supplemental information**

Table S1. Characterization of telomeric repeats in the haplomes of *Apera spica-venti*. Status indicates which ends of each chromosome telomeres are present. # left and # right indicate the number of repeats detected on each chromosome end. “a” and “b” are added to chromosome names to indicate haplome 1 (reference) and haplome 2 (alternate) haplotypes, respectively.

|  |  |  | Number of Telomere Repeats | |  |
| --- | --- | --- | --- | --- | --- |
| Chromosome | Length | Telomere Presence | Left | Right | |
| Chr01a | 678,048,256 | Both | 427 | 707 | |
| Chr02a | 629,761,991 | Both | 322 | 1,684 | |
| Chr03a | 606,585,553 | Both | 2,303 | 1,239 | |
| Chr04a | 586,252,376 | Left | 1,432 | N/A | |
| Chr05a | 540,854,188 | Left | 266 | N/A | |
| Chr06a | 549,308,307 | Both | 2,283 | 2,461 | |
| Chr07a | 471,366,042 | Right | N/A | 120 | |
| Chr01b | 668,678,507 | Both | 676 | 1,421 | |
| Chr02b | 626,881,108 | Both | 105 | 3,350 | |
| Chr03b | 593,747,648 | Both | 2,033 | 2,576 | |
| Chr04b | 574,205,699 | Left | 1,159 | N/A | |
| Chr05b | 538,259,495 | Left | 482 | N/A | |
| Chr06b | 529,946,436 | Right | N/A | 1,830 | |
| Chr07b | 462,709,132 | Left | 1,416 | N/A | |

Table S2. BUSCO results for the reference and alternative *Apera spica-venti* assemblies using the embryophyta_odb10 database as reference.

|  | Reference genome | Reference transcriptome | | Alternative genome | Alternative transcriptome |
| --- | --- | --- | --- | --- | --- |
| Complete | 1,599 (99.1%) | 1,452 (89.9%) | | 1,593 (98.7%) | 1,462 (90.6%) |
| Single-copy | 1,523 (94.4%) | 1,387 (85.9%) | | 1,525 (94.5%) | 1,401 (86.8%) |
| Duplicated | 76 (4.7%) | 65 (4.0%) | | 68 (4.2%) | 61 (3.8%) |
| Fragmented | 4 (0.2%) | 61 (3.8%) | | 5 (0.3%) | 50 (3.1%) |
| Missing | 11 (0.7%) | 101 (6.3%) | | 16 (1.0%) | 102 (6.3%) |
|  |  | | Total Searched:1,614 | | |

Table S3. Repeat content of each haplome estimated by RepeatModeler.

|  | Haplome 1 | Haplome 2 |
| --- | --- | --- |

| Type | Element # | Length | Genome % | Element # | Length | Genome % |
| --- | --- | --- | --- | --- | --- | --- |
| Retroelements: | 854,732 | 2.40 Gbp | 58.48% | 837,783 | 2.4 Gbp | 58.75% |
| SINEs: | 0 | 0 | 0% | 0 | 0 | 0% |
| Penelope | 1,284 | 212,868 | 0.01% | 1,330 | 222,876 | 0.01% |
| LINEs: | 54,252 | 52.0 Mbp | 1.26% | 53,114 | 51.0 Mbp | 1.27% |
| RTE/Bov-B | 1,249 | 985,198 | 0.02% | 1,218 | 927,495 | 0.02% |
| L1/CIN4 | 51,719 | 50.8 Mbp | 1.23% | 50,566 | 49.9 Mbp | 1.24% |
| LTR elements: | 800,480 | 2,358.1 Mbp | 57.22% | 784,669 | 2,316.7 Mbp | 57.49% |
| BEL/Pao | 1,006 | 544,947 | 0.01% | 1,059 | 566,266 | 0.01% |
| Ty1/Copia | 163,576 | 313.9 Mbp | 7.62% | 161,258 | 309.2 Mbp | 7.67% |
| Gypsy/DIRS1 | 624,817 | 2,025.3 Mbp | 49.14% | 611,643 | 1,989.2 Mbp | 49.36% |
| Retroviral | 11,081 | 18.3 Mbp | 0.44% | 10,709 | 17.8 Mbp | 0.44% |
| DNA transposons: | 129,403 | 133.1 Mbp | 3.23% | 126,179 | 129.3 Mbp | 3.21% |
| hobo-Activator | 17,269 | 9.2 Mbp | 0.22% | 17,057 | 8.7 Mbp | 0.22% |
| Tourist/Harbinger | 24,473 | 13.2 Mbp | 0.32% | 24,053 | 13.1 Mbp | 0.32% |
| Rolling-circles | 12,685 | 7.1 Mbp | 0.17% | 12,328 | 7.0 Mbp | 0.17% |
| Unclassified: | 1,430,592 | 919.7 Mbp | 22.32% | 1,398,955 | 892.3 Mbp | 22.14% |
| Total interspersed repeats: |  | 3.46 Gbp | 84.03% |  | 3.39 Gbp | 84.11% |
| Small RNA: | 1,551 | 8.5 Mbp | 0.21% | 1,039 | 5.0 Mbp | 0.12% |
| Simple repeats: | 187,944 | 8.1 Mbp | 0.20% | 183,585 | 7.9 Mbp | 0.2% |
| Low complexity: | 19,309 | 974,339 | 0.02% | 19,113 | 966,404 | 0.02% |

| Table S4. Information on *Apera spica-venti P450 genes.* | | | | | | | | | |  |
| --- | --- | --- | --- | --- | --- | --- | --- | --- | --- | --- |
|  | **No. of P450 per families** | **No. of P450 subfamilies** | **P450 subfamilies** | | **No. of P450 per subfamilies** | | **P450 fragments per subfamilies** | |  |  |
| **CYP51** | 22 | 2 | | G  H | | 2  20 | | -  7 | | |
| **CYP71** | 187 | 21 | | C  E  F  K  P  Q  R  S  T  U  V  W  X  Y  AA  AB  AD  AF  AK  AM  BU | | 13  12  32  16  3  1  8  1  5  1  3  5  17  32  12  10  2  3  7  1  3 | | 4  3  4  6  -  -  2  -  -  -  -  -  4  4  6  -  2  -  -  -  - | | |
| **CYP72** | 21 | 1 | | A | | 21 | | 5 | | |
| **CYP73** | 4 | 1 | | A | | 4 | | - | | |
| **CYP74** | 7 | 3 | | A  E  F | | 5  1  1 | | -  -  1 | | |
| **CYP75** | 6 | 2 | | A  B | | 1  5 | | -  3 | | |
| **CYP76** | 17 | 7 | | H  K  L  M  P  Q  V | | 8  1  2  3  1  1  1 | | 5  -  -  -  -  -  - | | |
| **CYP77** | 4 | 2 | | A  B | | 3  1 | | -  - | | |
| **CYP78** | 4 | 1 | | A | | 4 | | 1 | | |
| **CYP79** | 11 | 1 | | A | | 11 | | 2 | | |
| **CYP81** | 20 | 5 | | A  L  M  N  P | | 10  1  4  4  1 | | 2  2  -  - | | |
| **CYP84** | 8 | 1 | | A | | 8 | | 2 | | |
| **CYP86** | 4 | 2 | | A  E | | 3  1 | | -  - | | |
| **CYP87** | 8 | 2 | | A  B | | 2  6 | | -  2 | | |
| **CYP88** | 2 | 1 | | A | | 2 | | - | | |
| **CYP89** | 31 | 6 | | B  C  D  E  H  J | | 7  1  2  8  5  8 | | 4  -  -  -  1  - | | |
| **CYP90** | 2 | 2 | | A  D | | 1  1 | | -  1 | | |
| **CYP92** | 12 | 2 | | A  C | | 10  2 | | 2  - | | |
| **CYP93** | 8 | 2 | | F  G | | 1  7 | | -  2 | | |
| **CYP94** | 14 | 4 | | B  C  D  E | | 1  4  7  2 | | -  -  -  - | | |
| **CYP96** | 31 | 4 | | B  D  AB  E | | 26  2  2  1 | | 2  -  -  - | | |
| **CYP97** | 1 | 1 | | B | | 1 | | - | | |
| **CYP98** | 2 | 1 | | A | | 2 | | - | | |
| **CYP99** | 10 | 1 | | A | | 10 | | 4 | | |
| **CYP703** | 1 | 1 | | A | | 1 | | - | | |
| **CYP704** | 4 | 2 | | A  B | | 3  1 | | 1  - | | |
| **CYP707** | 2 | 1 | | A | | 2 | | - | | |
| **CYP709** | 20 | 3 | | C  E  J | | 6  4  10 | | 1  1  2 | | |
| **CYP710** | 1 | 1 | | A | | 1 | | - | | |
| **CYP711** | 4 | 1 | | A | | 4 | | - | | |
| **CYP714** | 5 | 3 | | B  C  D | | 2  2  1 | | -  -  - | | |
| **CYP715** | 1 | 1 | | B | | 1 | | - | | |
| **CYP721** | 2 | 1 | | B | | 2 | | 1 | | |
| **CYP722** | 1 | 1 | | B | | 1 | | - | | |
| **CYP723** | 12 | 4 | | A  B  H  C | | 6  4  1  1 | | -  -  -  - | | |
| **CYP724** | 1 | 1 | | A | | 1 | | - | | |
| **CYP727** | 1 | 1 | | A | | 1 | | - | | |
| **CYP728** | 15 | 2 | | A  B | | 3  12 | | 1  1 | | |
| **CYP729** | 2 | 1 | | A | | 2 | | 1 | | |
| **CYP733** | 1 | 1 | | A | | 1 | | - | | |
| **CYP734** | 3 | 1 | | A | | 3 | | - | | |
| **CYP735** | 1 | 1 | | A | | 1 | | - | | |
| **Total** | **513** | **102** | | **-** | | **513** | | **92** | | |


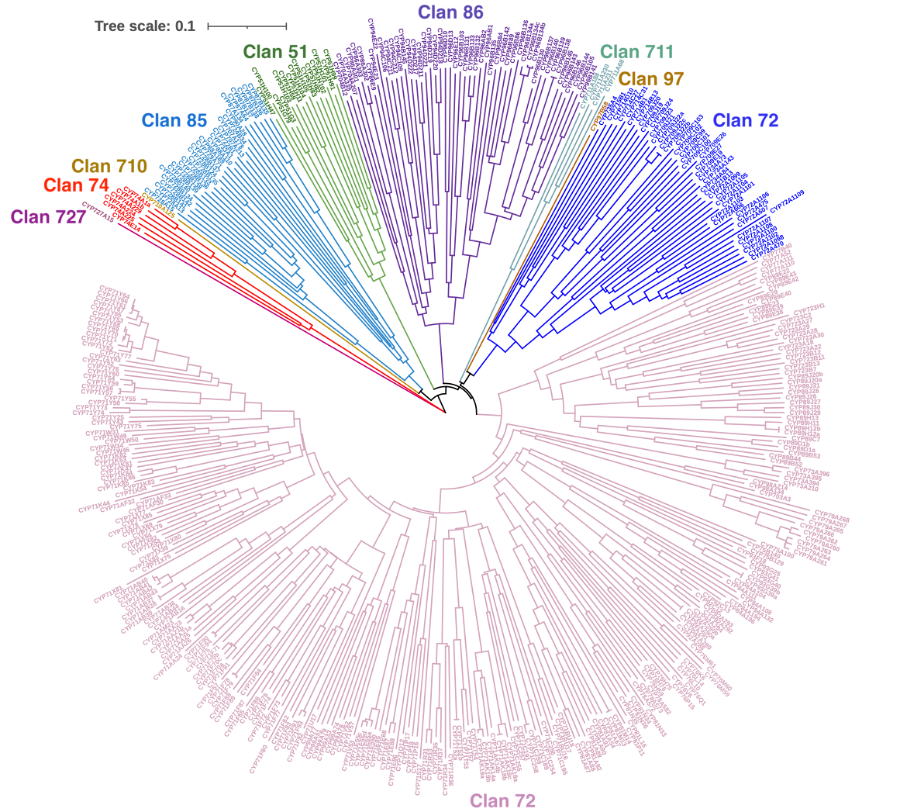


**Figure S1. Phylogenetic analysis of 421 full-length *Apera spica-venti* P450 genes.** The neighbor-joining (NJ) tree was generated using MEGA X software with 1,000 bootstrap replications. P450 gene clans are distinguished by different colors.


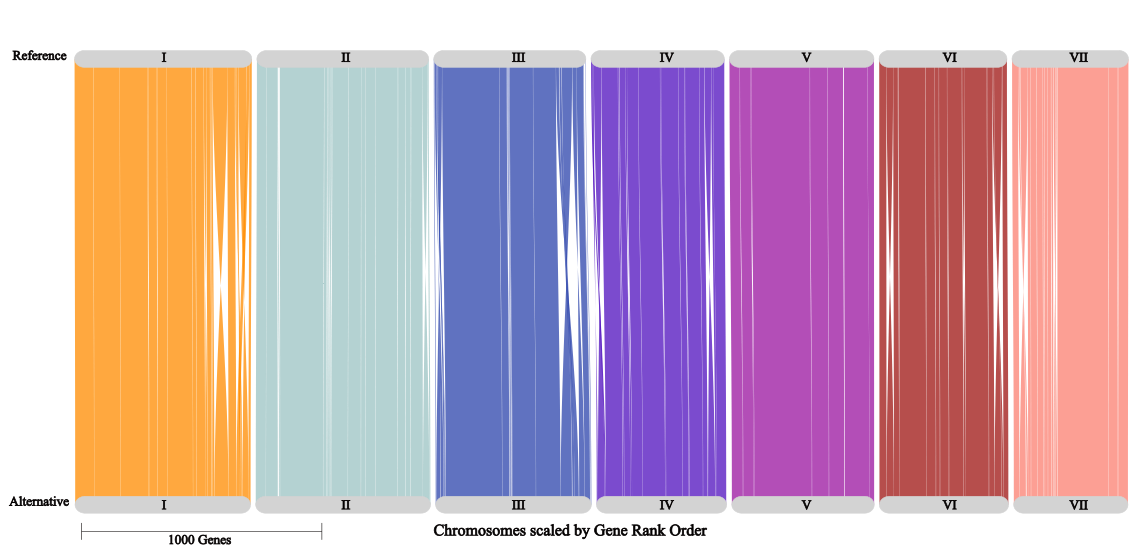


Figure S2. This plot shows synteny mapping between both assemblies demonstrating structural similarities between the haplotypes of *A. spica-venti* as generated via GENESPACE (Lovell et al. 2022) using translations from nucleotide to protein sequences (GFFRead). This figure demonstrates the degree of similarity between the two haplotypes across each assembly in that the majority of the two assemblies are structurally identical. Variation from this overall trend can be seen as large inversions in chromosomes 1, and 3, and smaller inversions in chromosomes 4, 6, and 7.
